# Supplementary material for: Association of Plasma Carotenoid and Malondialdehyde Levels with Physical Performance in Korean Adolescents
Source: Int J Environ Res Public Health. 2022 Apr 3;19(7):4296. doi: 10.3390/ijerph19074296 (PMC8998767; doi:10.3390/ijerph19074296)
Supplement: Supplementary file 1 [file ijerph-19-04296-s001.zip › ijerph-1624162-supplementary.pdf]

On-line Supplementary Material

Association of Plasma Carotenoid and Malondialdehyde Levels with Physical Performance in Korean Adolescents

Heeyeon Joo, Jiyoung Hwang, Ji Yeon Kim, Saejong Park, Hyesook Kim, and Oran Kwon

On-line Supplemental Table

Supplementary Table S1. Eating habits of Korean adolescents. [1]

	**Total (*n* = 381)**	**Boys (*n* = 164)**	**Girls (*n* = 217)**	***p***
Breakfast	313 (82.2)	137 (83.5)	176 (81.1)	0.5396
Fruits	370 (97.1)	158 (96.3)	212 (97.7)	0.4344
Fast foods	378 (99.2)	162 (98.8)	216 (99.5)	0.4068
Carbonated drinks	356 (93.4)	156 (95.1)	200 (92.1)	0.2486
Late-night snacks	320 (84.2)	142 (87.1)	178 (82.0)	0.1782

[1] Values are expressed as *n* (%). Breakfast was defined as eating once in the last week. Fruits, fast foods, carbonated drinks, and late-night snacks were defined as eating once in the last 30 days.

Supplementary Figure S1: HPLC elution profile of carotenoids in the plasma of Korean adolescents.

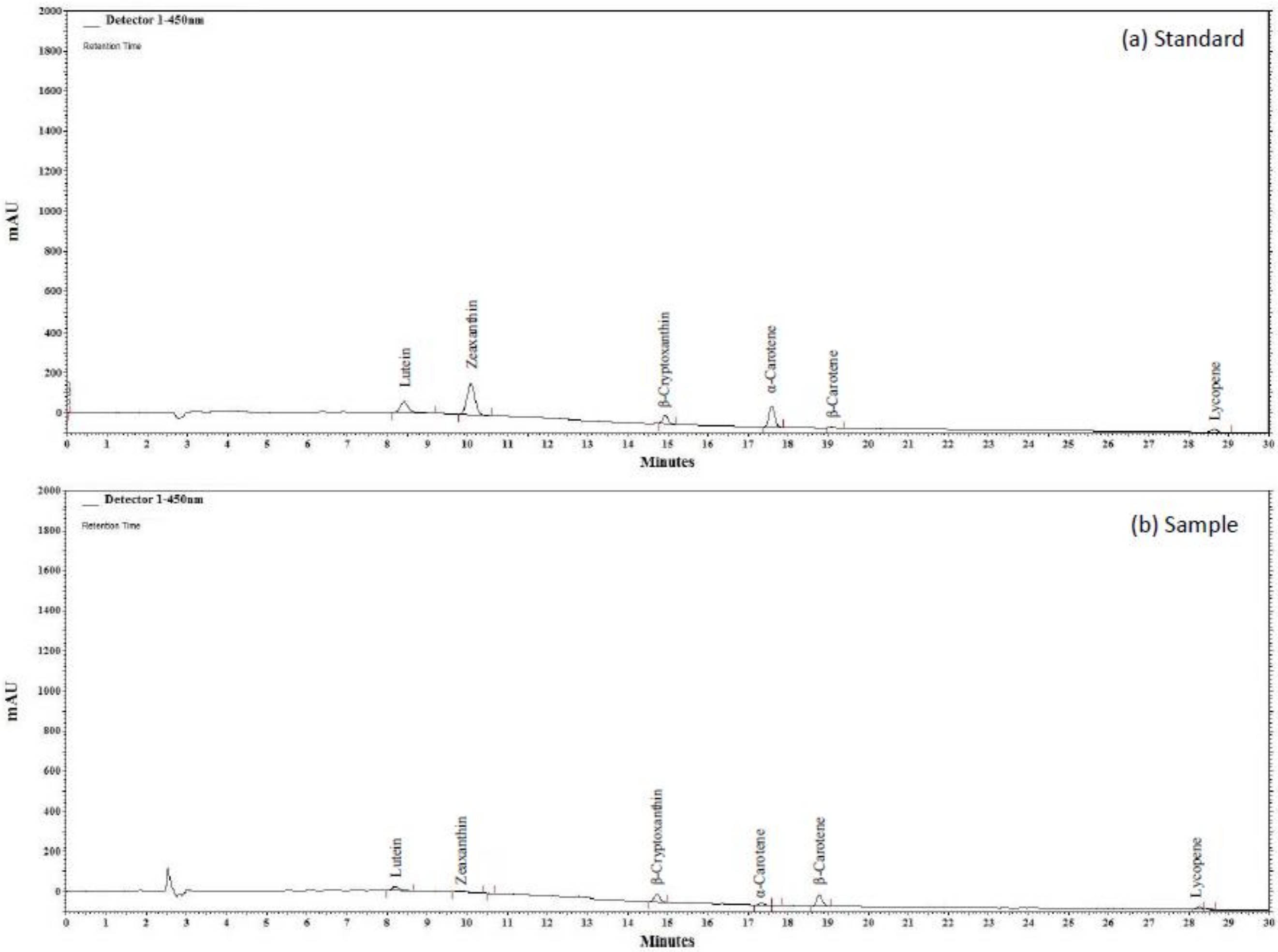

Supplementary Fig. S1: HPLC elution profile of carotenoids in the plasma of Korean adolescents. (a) Standard; (b) Sample.
